# Bone-Regenerative Ability of Platelet-Rich Plasma Following Sinus Augmentation with Anorganic Bovine Bone: A Systematic Review with Meta-Analysis

**DOI:** 10.3390/bioengineering9100597

**Published:** 2022-10-21

**Authors:** Eduardo Anitua, Mikel Allende, Asier Eguia, Mohammad Hamdan Alkhraisat

**Affiliations:** 1 Regenerative Medicine Department, BTI Biotechnology Institute, 01005 Vitoria, Spain; 2 Clinical Research, University Institute for Regenerative Medicine and Oral Implantology (UIRMI), 01005 Vitoria, Spain

**Keywords:** maxillary sinus floor elevation, platelet-rich plasma, platelet rich in growth factors, alveolar bone regeneration, histomorphometry

## Abstract

Background: The objective of this systematic review is to assess the effect of the adjuvant use of platelet-rich plasma (PRP) and its type on new bone formation by anorganic bovine bone during maxillary sinus floor augmentation procedure. Methods: PubMed, Cochrane Central Register of Controlled Trials, and Ovid databases were searched for relevant studies published up to 16 September 2021. Randomized clinical trials (RCTs) and non-randomized controlled clinical trials (CCTs) that reported data on the new bone formation (measured by histomorphometric analysis) were considered. Risk of bias and quality assessment of included studies were evaluated following the Cochrane Handbook for Systematic Reviews of Interventions and the Risk Of Bias In Non-randomised Studies of Interventions (ROBINS-I) tool. Strength of evidence was assessed following the approach of the Agency for Healthcare Research and Quality (AHRQ) through its evidence-based practice center (AHRQ EPC). The meta-analysis was based on the primary outcome of newly formed bone, for which the standard mean difference was calculated. Results: After the application of eligibility criteria, six clinical trials (three RCTs and three CCTs) covering 85 maxillary sinus floor elevation procedures were included. The pooled new bone formation value for PRP was 1.67 (95% CI: −0.15 to 3.49; I^2^: 86%), indicating the absence of significant effect. Plasma rich in growth factors (PRGF) was the pure PRP tested in five of the included studies. When sub-group (type of PRP) meta-analysis was performed, significantly higher new bone formation was observed in the PRGF group [2.85 (95% CI: 0.07 to 5.64; I^2^: 88%)] in comparison to the control group. Conclusions: A beneficial effect on new bone formation after maxillary sinus floor elevation can be obtained when anorganic bovine bone is mixed with PRGF.

## 1. Introduction

Placement of dental implants is the most frequent treatment option for oral rehabilitation of partially and totally edentulous patients [1,2,3]. Bone density and volume are of key relevance when planning implant-supported rehabilitation [4]. In this sense, the posterior maxilla can be considered a challenging area due to the low level of mineralization, the alveolar bone atrophy, and maxillary sinus pneumatization [5,6]. Consequently, the long-term implant survival rate in this area is reduced [7,8].

The maxillary sinus floor elevation (MSA) technique was introduced in the late 1970s to overcome these problems [9,10]. Using this method, bone grafts are placed in the maxillary sinus floor to increase bone volume and density [11]. Several studies have reported outcomes of good predictability and long-term efficacy [12,13,14,15]. 

In this procedure, different types of bone-grafting materials have been used to stabilize blood clotting under the Schneiderian membrane. Autogenous bone [16], allograft [17], xenograft [18], or alloplastic [19] bone substitutes [20] have been grafted in MSA procedures. These different biomaterials heal and mature differently, and show differences in their osteogenic properties (osteoconductivity, osteogenesis, osteoinductivity) [21]. Of the available bone substitutes, bovine-derived grafts have been widely used due to their biocompatibility, osteoconductivity, and stability [22,23].

Newly formed bone is considered a critical factor in the success of the bone augmentation procedures, and the use of biologic mediators has been proposed for its enhancement [24,25,26,27]. Platelet-rich plasma (PRP) is one of the biological alternatives that has attracted the attention of clinicians. This preparation is characterized by a higher platelet concentration than the peripheral blood. Once activated, platelets release their cargo of biomolecules and growth factors, and the plasmatic fibrinogen is converted to fibrin. Thus, a fibrin-based matrix enriched in growth factors and cytokines is obtained [28,29]. The use of PRP to regenerate tissues has been reported in several different medical fields, including traumatology [30], dermatology [31], ophthalmology, and oral and maxillofacial surgery [32] among others. However, as PRP-generating kits and protocols are highly heterogeneous [28], their collection under the same umbrella can complicate the interpretation of the results. One of the main differences between PRPs is the inclusion or otherwise of leukocytes. Two main types of PRP have been described: pure PRP lacking leukocytes (P-PRP) and leukocyte-rich PRP (L-PRP) [33,34]. 

The regenerative ability of PRPs has been recognized in terms of patient discomfort and soft-tissue healing when combined with bovine bone-grafting material [35]. However, the bone-regenerative potential of PRPs remains controversial; while some studies have reported favorable results [36,37,38,39], other studies reported no benefit in terms of new bone formation [8,35,40]. Furthermore, none of the published systematic reviews have assessed exclusively bovine bone-derived grafts or considered the heterogeneity of PRPs in the meta-analysis, making interpretation even more complex. The purpose of this systematic review with meta-analysis is to investigate the effects of the different classes of PRPs for bone regeneration in MSA when associated with grafting material of bovine origin.

## 2. Materials and Methods

### 2.1. Protocol Registration and Reporting Format

The present systematic review was designed following the guidelines of the 2020 Preferred Reporting Items for Systematic Review and Meta-Analysis (PRISMA) statement [41]. The protocol was registered and allocated in the PROSPERO database (CRD42021279012), hosted by the National Institute for Health Research, University of York, Center for Reviews and Dissemination. All amendments performed during the review process were registered indicating the date and the reason for the change.

#### Focus Question

The aim of this review was to address the following question:

What is the clinical efficacy of PRP in combination with anorganic bone grafts for bone regeneration in maxillary sinus floor elevation?

### 2.2. PICO Strategy

The following framework of population, intervention, comparison, and outcome was used:

- (P) Population: Randomized clinical trials (RCTs) and non-randomized clinical trials (CCTs) of patients requiring MSA.
- (I) Interventions: PRP in combination with anorganic bovine bone graft.
- (C) Comparison: Anorganic bovine bone graft alone.
- (O) Outcome: The primary outcome was new bone formation.

### 2.3. Eligibility Criteria

The inclusion criteria for this study were the following: RCT and CCT studies; studies performed in humans; bone grafting with bovine origin; histomorphometric evaluation of augmented bone change; anorganic bovine bone grafting without PRP as control. The exclusion criteria were the following: in vitro and animal studies; reviews or retrospective studies; and articles not published in the English language.

### 2.4. Data Sources and Search Strategy

**Figure 1:**
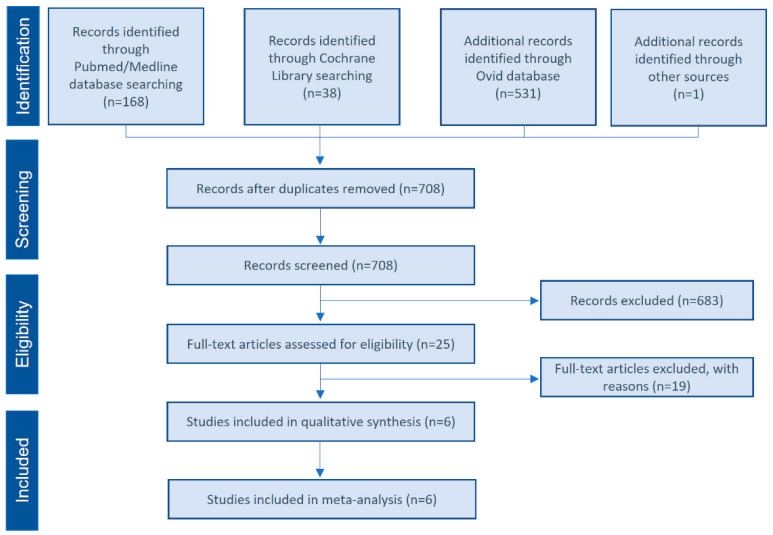
Selection flow diagram. PRISMA flow diagram of the screening and selection process.

A systematic search without applied filters was performed in MEDLINE/Pubmed, Cochrane Central Register of Controlled Trials, and Ovid electronic databases from inception to 16 September 2021. Only prospective controlled trials using a mixture of xenograft and PRP for maxillary sinus floor elevation procedures were considered. The following keywords were used in the search strategy: (bone graft OR allograft OR autogenous OR xenograft) AND (platelet rich plasma OR PRP) AND (sinus lift OR sinus elevation OR sinus bone graft OR sinus augmentation). In addition, clinical trial registries (http://www.clinicaltrials.gov) (accessed on 16 September 2021) were consulted to find studies in the gray literature. The search was restricted to the English language. Related systematic reviews were also assessed to identify possible additional studies. The search was limited to human studies. Figure 1 details the study selection flowchart. 

### 2.5. Data Collection and Management

Titles identified from the search were reviewed by one author (M.A.) to exclude any that did not examine the research question. Full texts of the remaining articles were reviewed independently by two authors (M.A. and A.E.) to identify studies that met all criteria for inclusion in the quantitative synthesis. Discrepancies were resolved by referring to the original publication. Studies that did not meet the inclusion criteria were excluded, and the reasons for exclusion are indicated in Supplementary Table S1.

### 2.6. Data Extraction

The relevant data of included studies were extracted into a pre-designed spreadsheet by one author (M.A.). The following characteristics of each study were extracted: (a) study characteristics—primary author, time of study, and year of publication; (b) study design characteristics—RCT, non-randomized controlled trial; (c) patient characteristics—age, sex, and number of augmented sinuses; (d) healing time; (e) outcome assessment—histology evaluation technique; (f) intervention groups—control and experimental groups. M.A. entered data into Review Manager 5.4, double checking it for accuracy.

### 2.7. Risk of Bias in Individual Research Studies

The methodological quality of RCT studies was evaluated by M.A. and M.H.A. using the risk-of-bias assessment tool outlined in the Cochrane Handbook for Systematic Reviews of Interventions [42]. In brief, the following criteria were classified as adequate (+), inadequate (−) or unclear (?): the method of randomization, allocation concealment, the blinding of participants and/or personnel, blinding of outcome assessment, incomplete outcome data, selective reporting, and other sources of bias. After these domains were assessed, the Cochrane risk-of-bias tool allowed production of an overall risk-of-bias classification of high, low, or unclear. An overall rating of low risk was assigned when none of the six domains were found to be at high risk and three or fewer domains were found to be at unclear risk. An overall rating of moderate risk was assigned when one domain was found to be at high risk, or no domains were found to be a high risk but four or more were found to be at unclear risk. In all other cases, the trial was classified as having a high overall risk of bias. Any discrepancies were resolved by open discussion between reviewers.

The risk of bias of the included prospective non-randomized controlled trials was assessed by M.A. and M.H.A., following the risk of bias in non-randomized studies on intervention (ROBINS-I) tool. This tool considers risk of bias due to confounding factors, selection of participants into the study, classification of interventions, deviations from intended intervention, missing data, measurement of outcomes, and selection of reported results [43]. Any discrepancies were resolved by open discussion between reviewers. 

### 2.8. Outcomes

The primary outcome was new bone formation of the augmented area of the sinus floor, measured by histomorphometry analysis. New bone formation was defined by the respective study authors; if this was not clearly defined, it was classified based on the presence of mineralized tissue.

### 2.9. Statistical Analysis

The software Review Manager 5.4 (The Nordic Cochrane Centre, Copenhagen, Denmark) was employed to perform the meta-analysis. New bone formation was assessed as continuous outcome variables by the inverse variance method, and recorded as the standard mean difference (SMD) with 95% confidence interval (CI). Pooled SMD was interpreted as follows [44]: a small effect at 0.2, a medium effect at around 0.5, and a large effect at ≥0.8. If the 95% CI did not include the value 0, then the pooled SMD was statistically significant (*p* < 0.05). 

Heterogeneity among the selected studies of treatment effect was assessed using the I^2^ statistic, with values over 50% indicating substantial heterogeneity [45]. When no significant heterogeneity was found, the fixed-effects model was used; the random-effects model was adopted when significant heterogeneity was detected. The results were displayed in a forest plot to provide a graphical overview of the data. Where possible, a sub-group analysis was performed according to the type of PRP used (L-PRP or P-PRP). Meta-analyses were performed only for articles with similar outcome measures at comparable observation times. Due to the low number of studies, sensitivity analyses and assessment of reporting biases by funnel plot could not be performed. 

### 2.10. Strength of Evidence

The strength of evidence (SoF) of selected studies was assessed by two reviewers (M.A. and A.E.) applying the AHRQ EPC approach (Evidence-based Practice Center; US Agency for Healthcare Research and Quality) for comparative effectiveness reviews (CERs) [46]. Any discrepancies were resolved by open discussion between reviewers. In cases of disagreement, consensus was reached by open discussion.

## 3. Results

### 3.1. Study Selection and Characteristics

Figure 1 shows the flow diagram of study selection. A total of 708 studies were screened. Of these, 683 publications were excluded after the application of the inclusion and exclusion criteria. The remaining 25 articles were considered potentially relevant and were evaluated by reading the full texts. Of these, six studies were considered for qualitative and quantitative analysis [8,35,36,38,40,47], while 19 articles were excluded (Supplementary Table S1). 

The main characteristics of the included studies are summarized in Table 1. Three were RCTs and three used prospective controlled designs without randomization. A total of 85 maxillary sinus floor elevation procedures were performed. Participants’ mean ages ranged from 48 to 72 years. Five studies used a split-mouth design, whereas one study included bilateral and unilateral sinuses. Table 2 describes the primary outcomes of new bone formation.

### 3.2. Risk of Bias of Included Trials

**Figure 2:**
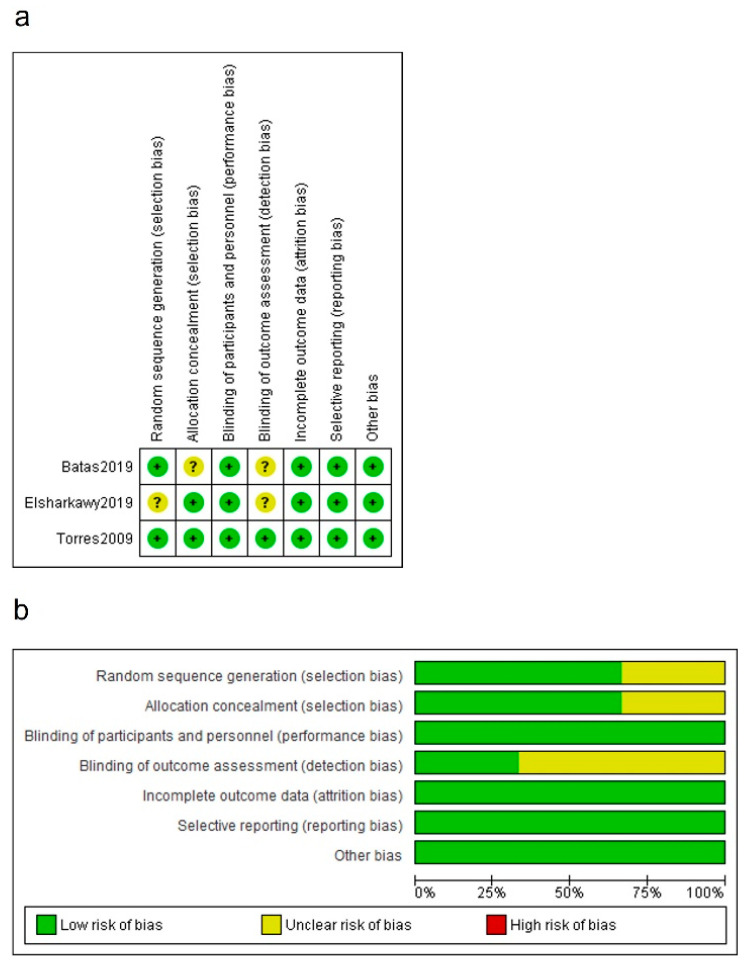
Quality assessment of the included RCTs. (**a**) Risk of bias summary, review of authors’ judgments about each risk-of-bias item for each included study. (+): low risk of bias. (?): unclear risk of bias. (**b**) Risk-of-bias graph, review of authors’ judgments about each risk-of-bias item, presented as percentages across all included studies [36,38,40].

Evaluation of the risk of bias for the included RCTs is depicted in Figure 2. Two studies clearly addressed the procedure for the allocation concealment and were scored as low risk of bias, while the other publications did not provide this information. Although it was not possible to blind the personnel, all studies were scored as low risk of bias due to the outcome characteristics. One trial described the blinding procedure in the outcome evaluation process and was scored as low risk of bias. Other trials were considered as unclear risk of bias, as this data could not be found in the texts. The data were gathered for all the included studies. After the assessment, all the RCTs were classified as low risk of bias.

**Figure 3:**
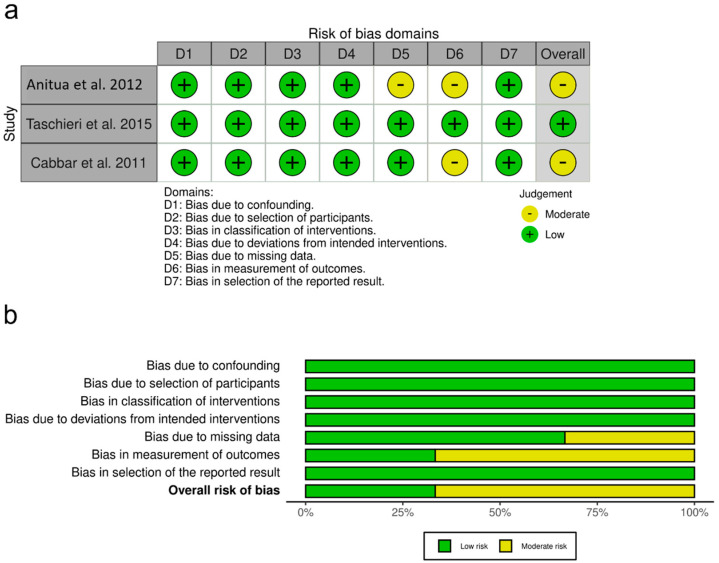
Quality assessment of the included prospective non-randomized studies. (**a**) Risk-of-bias summary, review of authors’ judgments about each risk-of-bias item for each included study. (+): moderate risk of bias. (**b**) Risk of bias graph, review of authors’ judgments about each risk-of-bias item, presented as percentages across all included studies [8,35,47].

The risk-of-bias assessment of non-randomized studies assessed using the ROBINS-I tool is summarized in Figure 3. Two non-randomized trials were scored as having an overall moderate risk of bias, whereas one trial was judged as low risk of bias. 

### 3.3. Information from All Included Studies

**Figure 4:**
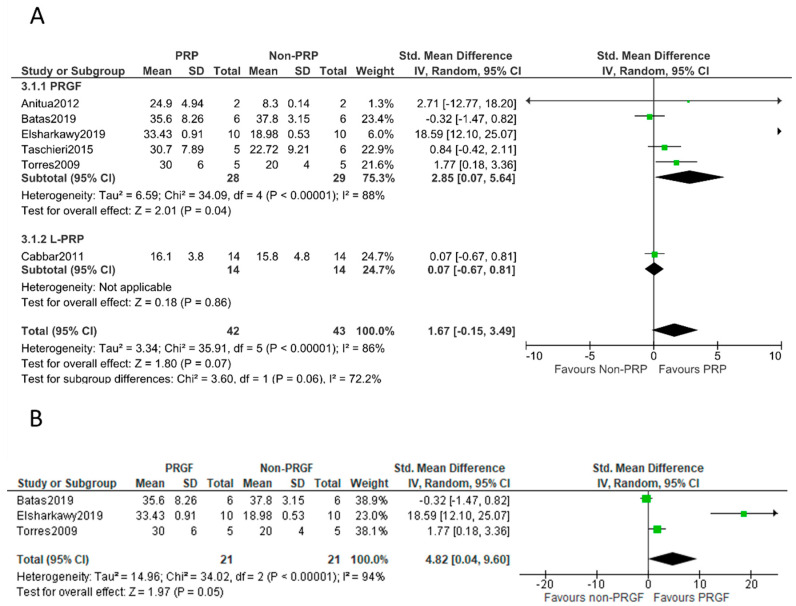
Meta-analysis of the studies evaluating new bone formation. (**A**) Randomized clinical trials and controlled clinical trials. (**B**) Only randomized clinical trials. SE: standard error. SMD: standardized mean difference. CI: confidence interval [35,36,38,40,47].

Six controlled trials evaluated the addition of PRPs to bovine bone-grafting material in sinus-lifting procedures. A total of 85 sinuses were included (42 and 43 for PRP and non-PRP groups, respectively). All the biopsies were harvested five to six months after surgery and stained with basic fuchsine and methylene blue [36], van Gieson’s picro-fuchsin [40], Masson trichrome special stain [38], Alcian blue, or hematoxylin [35,47]. Considering the pooled data, the meta-analysis found no significant differences in the primary outcomes of newly formed bone when comparing the PRP group to the control group (SMD: 1.67; 95% CI: −0.15 to 3.49). When a subgroup analysis was performed, a significant effect of P-PRP was observed (SMD: 2.85; 95% CI: 0.07 to 5.64). In all these studies, PRGF was applied. L-PRP showed no significant effect (SMD: 0.07; 95% CI: −0.67 to 0.81) (Figure 4A).

### 3.4. Information from Randomized Clinical Trials

Three RCTs evaluated the effect of PRP on new bone formation; all these studies employed PRGF. Bone biopsy specimens were harvested at 6 months post-operation and were stained with basic fuchsine and methylene blue [36], van Gieson’s picro-fuchsin [40], or Masson trichrome special stain [38]. The meta-analysis of these RCTs confirmed higher new bone formation in the PRGF group (SMD: 4.82; 95% CI: 0.04 to 9.60) (Figure 4B).

### 3.5. Strength of Evidence

Overall evidence was qualified using the AHRQ EPC approach [46] for RCTs and non-randomized controlled trials. Initial ratings were considered high, based on the type of studies, low risk of bias, and directness. Although consistency in the direction of effect was observed, high heterogeneity in effect size precluded declaration of consistency. The latter and the sample size led to downgrading of the overall SoF to moderate. A summary of the findings can be found in Table 3.

## 4. Discussion

The use of PRPs to enhance postoperative tissue healing has been reported in many studies [28,39,48,49,50]. However, it remains unclear whether the adjuvant use of PRP could enhance new bone formation after alveolar ridge augmentation. This systematic review aims to assess the evidence from RCTs and prospective controlled trials that tested the adjuvant use of PRP with anorganic bovine bone in MSA procedures. 

The wide range of available protocols for PRP preparation has frequently led to autologous preparations that differ significantly in composition and function [28]. P-PRP is classified as a type of platelet-rich plasma characterized by the absence of leukocytes [34]. PRGF, a type of P-PRP, was tested in five of the included studies. It is prepared following a reference protocol that has undergone no major modifications since its introduction [51], facilitating the interpretation and reproducibility of the clinical data. It is plausible that the manufacturing methods and characteristics of PRPs may have influenced outcomes in bone grafting [52,53,54]. To date, published systematic reviews have not considered the type of PRP in their analysis, and conflicting results have been reported. In 2011, Bae et al. [55] reported a beneficial effect of PRP on bone formation. In contrast, two other systematic reviews with meta-analysis found no beneficial effect of mixing PRP with bone-grafting material [56,57]. These conflicting results may be explained by the heterogeneity among the PRPs assessed in the respective studies. Adequate characterization and classification of PRP preparations is needed to better assess the evidence about mixing PRP with biomaterials for bone regeneration. Sub-group analysis (P-PRP and L-PRP) can provide new insights into the bone-regenerative ability of PRP in MSA. For that purpose, this systematic review has aimed at assessing the bone-regenerative potential of different PRP types when combined with bovine bone-grafting materials.

The results of this meta-analysis have shown the importance of assessing the influence of the type of PRP on new bone formation after MSA with anorganic bovine bone. The effect of PRP (including all types) was not statistically significant in terms of enhancing new bone formation. However, sub-group analysis shows that PRGF (P-PRP) mixed with anorganic bovine bone resulted in higher new bone formation (the effect was statistically significant). This beneficial effect of PRGF could be related to the decrease in tissue inflammation and to the content of biological mediators [33,47]. The fibrin of the PRGF supports cellular function (adhesion, spreading, and proliferation) and serves as a transitional scaffold for the development of the provisional matrix that directs tissue healing [29]. Recently, it was shown that while PRGF supported the proliferation of cells (fibroblasts and osteoblasts) and the synthesis of extracellular matrix proteins, L-PRP inhibited these functions [29]. Fibrin also stimulates angiogenesis by stimulating the spread and proliferation of endothelial cells and the formation of capillary tubes [58]. PRGF also progressively releases growth factors and cytokines to induce healing [29]. It stimulates osteoprogenitor cells and osteoblasts through the action of several growth factors such as transforming growth factor-β (TGF-β), insulin-like growth factor (IGF-I), and platelet-derived growth factor (PDGF) [58]. Unfortunately, data for L-PRP are limited and we found only one relevant prospective study [8]. Torres et al. conducted an RCT to assess P-PRP and anorganic bovine bone in MSA [36]. The outcomes indicated higher new bone formation in the P-PRP group. Similarly, Anitua et al. in a split-mouth prospective study described higher new vital bone growth in the P-PRP group [47]. In recent years, two other RCTs have been published with contradictory results [38,40]. In the study published by Batas et al. no differences were observed regarding new bone formation [40], while Elsharkawy et al. observed a significant increase favoring the use of P-PRP [38]. These findings must be interpreted with caution due to the small number of participants included in each trial and the high heterogeneity between the studies. 

The type of bone-grafting material is another factor that can influence new bone formation. In a recent systematic review, Stumbras et al. [59] identified autologous bone as the best-performing graft for inducing new bone formation after MSA. In fact, autologous bone is still considered the gold standard in bone regeneration due to its high capacity for osteoconduction, osteoinduction, and osteogenesis [60]. However, there remain concerns about patients’ discomfort and potential complications at donor sites. Autologous bone has also shown high resorption and less volume gain [60]. Other bone-grafting materials that offer higher stability have been used, such as anorganic bovine bone. Consequently, heterogeneity of grafting is another confounding factor that should be considered when assessing the adjuvant use of PRP in MSA. Accordingly, our systematic review has been restricted to anorganic bovine bone to allow more homogenous data content. Four of the selected studies employed Bio-Oss as grafting material, while the other two described the use of other commercially available bovine bone derivatives (SmartBone and Unilab Surgibone) in the augmented sinus. Interpretation of the meta-analysis results is valid only for grafting material of bovine origin; future studies should be designed to clarify the respective performances of P-PRP and L-PRP with other bone graft types. 

Furthermore, it is believed that preoperative residual bone height may influence bone regeneration and implant success rate after MSA [61,62]. There was high heterogeneity regarding residual bone height (RBH) in the selected articles. One study included sinuses with RBH less than 7 mm, while other study only recruited patients with RBH of 1 to 3 mm. A further study did not provide information regarding RBH.

The strengths of this systematic review include a comprehensive literature search, restriction to bovine bone-grafting material to increase homogeneity, a robust methodology to perform quantitative synthesis of data, assessment of bias using the Cochrane Handbook for Systematic Reviews of Interventions, and evaluation of the SoF with the AHRQ EPC approach [46]. However, several limitations should be acknowledged. First, there was only one study in the L-PRP group, therefore, the conclusions for this type of platelet preparation should be interpreted with particular caution. Second, this systematic review included trials with small sample sizes (four out of six selected trials included fewer than 10 sinuses per group) and high heterogeneity among studies. Third, differences of residual bone height between studies might be an important source of bias. In addition, half of the included studies were non-randomized prospective studies, which also might lead to bias. However, this limitation was overcome with a separated meta-analysis that included RCTs exclusively. Language restriction to English was a further limitation of this systematic review.

## 5. Conclusions

Despite the limitations of the included studies, this systematic review with meta-analysis revealed a beneficial effect on bone formation in MSA when bovine bone-grafting material was mixed with PRGF. However, the number of studies was low and further RCTs are needed. 

## Figures and Tables

**Table 1:** Main characteristics of the included studies.

Study	Design	PRP Type	Patients (Sinus)	Sex (F/M)	Age (Years)	RBH (mm)	Healing Time (Months)	Graft Material	
								Control	Test
Torres et al., 2009 [36]	Split mouth RCT	P-PRP	87 (144)	47/40	52–78	<7	6	Bio-Oss	Bio-Oss + PRGF
Batas et al., 2019 [40]	Split mouth RCT	P-PRP	6 (12)	NR	NR	<3	6	Bio-Oss	Bio-Oss + PRGF
Elsharkawy et al., 2019 [38]	RCT	P-PRP	25 (30)	7/18	55 ± 7	<5	6	SmartBone	SmartBone + PRGF
Taschieri et al., 2015 [35]	Split mouth CCT	P-PRP	6 (12)	4/2	48–71	<4	6	Bio-Oss	Bio-Oss + PRGF
Anitua et al., 2012 [47]	Split mouth CCT	P-PRP	5 (10)	NR	NR	1–3	5	Bio-Oss	Bio-Oss + PRGF
Cabbar et al., 2011 [8]	Split mouth CCT	L-PRP	10 (20)	3/7	53.7 ± 0.8	NR	6	Unilab Surgibone	Unilab Surgibone + L-PRP

RCT: Randomized clinical trial. CCT: Controlled clinical trial. P-PRP: pure platelet-rich plasma. L-PRP: leukocyte platelet-rich plasma. RBH: Residual bone height. PRGF: Plasma rich in growth factors.

**Table 2:** Histomorphometric analysis of the included studies.

Study	Time of Measurement (Months)	Sample Size	Staining	Histomorphometric Analysis (%)	
		C/T		Control	Test
Torres et al., 2009 [36]	6	5/5	Basic fuchsine and methylene blue	20 ± 4	30 ± 6
Batas et al., 2019 [40]	6	6/6	van Gieson’s picro-fuchsin	37.8 ± 3.15	35.6 ± 8.26
Elsharkawy et al., 2019 [38]	6	10/10	Masson trichrome special stain	18.98 ± 0.53	33.43 ± 0.91
Taschieri et al., 2015 [35]	6	6/5	Alcian blue and hematoxylin	22.72 ± 9.21	30.7 ± 7.89
Anitua et al., 2012 [47]	5	2/2	Alcian blue and hematoxylin-eosin	8.3 ± 0.14	24.9 ± 4.94
Cabbar et al., 2011 [8]	6	14/14	Toluidine blue	15.8 ± 4.8	16.1 ± 3.8

**Table 3:** PRP vs. Non-PRP strength of evidence. Summary of findings.

Number of Studies; Subjects	Domains Pertaining to Strength of Evidence				Magnitude of Effect and Strength of Evidence
	**Risk of Bias**	**Consistency**	**Directness**	**Precision**	**Std. Mean Difference**
**New Bone Formation (PRP vs. Non-PRP)**					**Moderate SoE**
6; 57	RCT(3)/CCT(3) LOW	Inconsistent	Direct	Precise	2.85; 95% CI: 0.07 to 5.64

## Data Availability

Data supporting the findings are available within the article and from the auKassoliss upon request.

## Supplementary Materials

The following supporting information can be downloaded at: https://www.mdpi.com/article/10.3390/bioengineering9100597/s1, Table S1: List of excluded studies with reasons for exclusion (References [63,64,65,66,67,68,69,70,71,72,73,74,75,76,77,78,79,80] are cited in the supplementary materials).
