# Supplementary material for: Bone-Regenerative Ability of Platelet-Rich Plasma Following Sinus Augmentation with Anorganic Bovine Bone: A Systematic Review with Meta-Analysis
Source: Bioengineering (Basel). 2022 Oct 21;9(10):597. doi: 10.3390/bioengineering9100597 (PMC9598686; doi:10.3390/bioengineering9100597)
Supplement: Supplementary file 1 [file bioengineering-09-00597-s001.zip › bioengineering-1943477-supplementary.pdf]

bioengineering

Systematic Review

Bone-Regenerative Ability of Platelet-Rich Plasma following Sinus Augmentation with Anorganic Bovine Bone: A Systematic Review with Meta-Analysis

Eduardo Anitua [1,2,*], Mikel Allende [1,2], Asier Eguia [2] and Mohammad Hamdan Alkhraisat [1,2]

1 Regenerative Medicine Department, BTI Biotechnology Institute, 01005 Vitoria, Spain
2 Clinical Research, University Institute for Regenerative Medicine and Oral Implantology (UIRMI), 01005 Vitoria, Spain
* Correspondence: eduardo@fundacioneduardoanitua.org; Tel.: +34-945-16-06-53

Table S1. Excluded studies (with reasons).

Study	Reason for exclusion
Yilmaz et al. 2013 [63]	New bone information is absent
Schaaf et al. 2007 [64]	Autologous bone graft
Schaaf et al. 2008 [65]	Autologous bone graft
Badr et al. 2016 [66]	Autologous bone graft
Khairy et al. 2012 [67]	Autologous bone graft
Raghoebar et al. 2004 [68]	Autologous bone graft
Consolo et al. 2007 [69]	Allograft
Karaca et al. 2017 [70]	New bone information is absent
Thor er al. 2005 [52]	Autologous bone graft
Thor et al. 2007 [71]	Autologous bone graft
Kassolis et al. 2005 [72]	Allograft
Witfang et al. 2003 [73]	Synthetic bone graft
Aimetti et al. 2009 [74]	Autologous bone graft
Lindeboom et al. 2007 [75]	Autologous bone graft
Poeschl et al. 2012 [76]	Algae-derived graft
Velich et al. 2004 [77]	Synthetic bone graft
Del Fabbro et al. 2013 [78]	New bone information is absent
Cömert et al. 2017 [79]	Synthetic bone graft
Cömert et al. 2016 [80]	Synthetic bone graft
